# Functional Foveal Splitting: Evidence from Neuropsychological and Multimodal MRI Investigations in a Chinese Patient with a Splenium Lesion

**DOI:** 10.1371/journal.pone.0023997

**Published:** 2011-08-26

**Authors:** Benyan Luo, Chunlei Shan, Renjing Zhu, Xuchu Weng, Sheng He

**Affiliations:** 1 Department of Neurology, First Affiliated Hospital, Zhejiang University School of Medicine, Hangzhou, China; 2 Department of Rehabilitation Medicine, First Affiliated Hospital of Nanjing Medical University, Nanjing, China; 3 Department of Neurology, Zhongshan Hospital, Xiamen University, Xiamen, China; 4 Key Laboratory of Mental Health, Institute of Psychology, Chinese Academy of Sciences, Beijing, China; 5 The Center for Human Brain Research, Hangzhou Normal University, Hangzhou, China; 6 Department of Psychology, University of Minnesota, Minneapolis, Minnesota, United States of America; Beijing Normal University, China

## Abstract

It remains controversial and hotly debated whether foveal information is double-projected to both hemispheres or split at the midline between the two hemispheres. We investigated this issue in a unique patient with lesions in the splenium of the corpus callosum and the left medial occipitotemporal region, through a series of neuropsychological tests and multimodal MRI scans. Behavioral experiments showed that (1) the patient had difficulties in reading simple and compound Chinese characters when they were presented in the foveal but left to the fixation, (2) he failed to recognize the left component of compound characters when the compound characters were presented in the central foveal field, (3) his judgments of the gender of centrally presented chimeric faces were exclusively based on the left half-face and he was unaware that the faces were chimeric. Functional MRI data showed that Chinese characters, only when presented in the right foveal field but not in the left foveal field, activated a region in the left occipitotemporal sulcus in the mid-fusiform, which is recognized as visual word form area. Together with existing evidence in the literature, results of the current study suggest that the representation of foveal stimuli is functionally split at object processing levels.

## Introduction

There are two competing theories regarding the cortical representation of the foveal vision, which is crucially important for many visual tasks such as reading and face recognition. The first theory, often referred to the “bilateral projection theory” (BPT), proposes that the foveal information from the left and right visual fields (LVF and RVF) overlaps along the vertical meridian and two complete copies of a foveally presented visual stimulus are projected in parallel to the early visual cortex of each hemisphere. This theory is supported by a number of physiological and anatomical studies in laboratory animals [1]–[7], as well as behavioral studies in hemianopia patients showing macular or foveal sparing and in commissurotomized patients showing better performance for foveal presentation than para-foveal/peripheral presentation in tasks relying on the integration of left and right visual field information [8]–[14]. More recently, Marzi et al. indicated bilateral representation of the fovea in healthy individuals by using the Poffenberger paradigm and monocular vision [15]. In a series of carefully designed experiments, Jordan et al. [16]–[20] reported negative evidence for a functional division in hemispheric processing at foveal field. In addition, some results of functional brain imaging studies also appear to be consistent with the BPT [21]–[23].

The second theory, often called the “split-fovea theory” (SFT), states that each half of the fovea is divided precisely at its vertical midline and visual information is exclusively projected to the contralateral visual cortex [24]–[48]. The most important line of evidence supporting this theory comes from studies on visual word processing. These studies showed that functional hemispheric differences, which used to be demonstrated by parafoveal presentation, can also be readily observed in foveal presentation conditions with a variety of experimental manipulations [34], as indicated by the optimal viewing position effect [24]–[25], [31], the word length effect [32], [37], case alternation effects [49], and the orthographic neighborhood effect [27], . Most recently, following the SERIOL model of visual word recognition [50], Haegen & Brysbaert provided further evidence for SFT by investigation of interhemispheric inhibition in the processing of visual word integration [51].

It should be noted that many of the studies on foveal representations are conducted in healthy subjects or patients with functionally and anatomically intact corpus callosum, which could make it difficult to make straightforward inferences about foveal representation in the two hemispheres. For example, there is the possibility that, even if there are bilateral projections (thus double cortical representations for foveal information), which the BPT assumes, the ipsilateral representation could be subordinated to the contralateral one and behavioral manifestation might be dominated or masked by contralateral information through corpus callosum inhibition [52]–[53] (see review of Bloom & Hynd [54]). On the other hand, even if the information in one half foveal field is initially projected only to the contralateral visual cortex as the SFT assumes, it could still be received slightly later by the ipsilateral visual cortex through quick splenium transfer. This effect is too fast to detect in many experimental conditions, except in some carefully designed experiments [55]–[58]. Therefore, subjects with disconnected communications between posterior hemispheres, such as patients with splenium lesion or commissurotomy, provide a unique opportunity to tease apart mixed accounts for cortical representations of foveal vision. Sieroff & Lavidor adopted reading tasks to test a patient with left medial occipital lesion which the author suggested to injure the splenium, and found evidence supporting SFT [59]. Unfortunately the authors did not use relatively direct imaging methods such as high resolution structural image or DTT (diffusion-tensor tractography) to confirm the disruption of the splenium. And, the words or pseudowords presented in LFF or RFF subtended a visual angle of 2 degree, larger than the maximum size of one half of foveal field (the central fovea is 1°–3°[2]–[3], [10], [29], half fovea should be 0.5°–1.5°).

In the present study, we studied a patient with a lesion in the splenium to explicitly test the BPT and SFT, using various experimental manipulations and with combined use of high resolution structural and functional MRI (fMRI), and diffusion tensor imaging (DTI) techniques. Multimodal MRI data would allow us to perform a detailed lesion analysis, by which the loci and degrees of lesions in both the gray and white matter of the patient's brain could be accurately evaluated.

Another merit of this study is that the patient was premorbidly a skilled Chinese reader. The majority of Chinese characters are left-right structured compounds, with one component on the left and the other on the right. There are two types of left-right structured compound characters. About 90% are semantic- phonetic characters (SP), in which the left component indicates the meaning of the whole character, whereas the right one provides a cue to the pronunciation of the character. About 10% of left-right structured compound characters are phonetic-semantic characters (PS), in which the left component provides phonetic information about the character and the right suggests the character's meaning. In both SP and PS, the semantic and phonological relationship between a character and the components that form this character is often subtle and not always reliable [41]. It should be noted that the stroke number of semantic components is typically smaller than that of phonetic components and the ratio of SP to PS character types is about nine to one. In other words, the left and right components in a character could remarkably differ in terms of visual complexity, occurrence frequency, and information density, which consequently may influence readers' attention and perception to the left and right components. In the behavioral Experiment 1, we used the same number of SP and PS characters (30 for each type) to control for the possible confounding. Furthermore, the semantic and phonetic components themselves are often simple characters with their own pronunciation and meaning [60]–[62]. These features make Chinese characters particularly suitable for addressing whether or not visual information is fovea-split, because the left and right components can be presented precisely within the left and right foveal field (LFF and RFF) respectively and tested separately at the component level or jointly at the whole character level [40]–[42]. Thus to test foveal representation in the brain, Chinese characters have an advantage over alphabetic words. In the latter there is often no clear (visual, semantic, or phonological) boundary between the left and right parts within a written word, particularly the short-length word consisting of only a few letters (unfortunately, such words are commonly used in previous studies).

Furthermore, because a Chinese character occupies a constant square-shaped space with the approximately same size, a whole character can be presented completely within a half foveal field (the visual angle of a character in normal reading texts is about 0.5° or less in both horizontal and vertical dimensions). This allows us to test the patient's reading performance in a variety of presentation conditions. We predict that, if the SFT is correct, our patient would be unable to correctly read the Chinese characters presented either in the central or in the LFF. This is because the information from the LFF (left components in the central condition or whole characters presented in the LFF), according to the SFT, is exclusively projected to the right visual cortex and cannot be transferred to the visual word form area (VWFA), a critical region for visual word recognition in the left lateral mid-fusiform [63]–[65]. If the BPT is correct, however, he should be able to read the foveally presented characters regardless which field the stimuli are presented, because all visual information of the presented character can be transferred to both hemispheres according to this theory.

Critically, for a good theory that genuinely describes the organizations and computations of the visual system, it must apply to a wide range of visual processes, not limited to reading tasks (See [30], [35] for more detailed discussion). We thus carefully designed a face recognition task, in which the patient was asked to make gender judgment while a chimeric face (composed of a half male face and a half female face) was presented entirely within the foveal field, with half face in the LFF and another half in the RFF. Because face recognition relies more on the right hemisphere [66]–[71] and the patient's left fusiform was damaged, we predict that, if the SFT is correct, his judgment would exclusively be based on the information from the half face left of the fixation. In contrast, if the BPT is correct, the information of both parts of the chimeric face would be sent to the fusiform face area (FFA) in the right fusiform gyrus [69], [72], and the patient would be able to see that the faces are chimeric.

Finally, to measure brain responses to the visual stimuli presented to the left or right regions within the foveal field, thus offering a more direct test for the BPT and SFT, we further conducted an fMRI experiment, in which Chinese characters were presented in the LFF or RFF. The SFT would predict that stimuli presented in the LFF activate the right early visual cortex while the left early visual cortex and the VWFA in the left fusiform would not be activated, due to the interruption of the splenium. In contrast, the BPT would predict that the early visual cortex in both hemispheres, as well as the VWFA in the left fusiform cortex, would be significantly activated regardless of LFF or RFF presentation of stimuli.

## Methods

### Case description

The patient was a right-handed Native Chinese, male and 80 years old, with 16 years of education. He had a stroke with a sudden onset of a blurred vision and light numbness over his left limbs in July of 2003. At that time he also complained about reading difficulties. Common therapies for stroke patients were administered following the first month of his stroke onset. Thereafter, he stayed in a sanitarium and regularly took oral medicines. In February of 2007, he was admitted to the 1^st^ affiliated hospital of Nanjing Medical University and complained of reading problem for about three years post-onset. Neurological examination revealed a right homonyous hemianopsia. The MRIs (January, 2004) showed that he had lesions involving the left occipitotemporal cortex and the left splenium of the corpus callosum (for details, see Lesion Analysis below). Neuropsychological assessments showed that the patient was unable to identify the left component of Chinese characters, a symptom similar to the cases reported by Binder et al. [73] in alphabetic readers (the authors labeled this symptom as left hemiparalexia). The patient often misread Chinese character as another one that shares the same right component as the target. For example, he misread 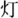 (/deng 1/, lamp) as 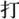 (/da 3/, beat), and 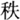 (/zhi 4/, order) as 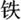 (/tie 3/, iron). Visual perimetry confirmed a right homonymous hemianopia but with 1.5° of foveal sparing. The Line Bisection Test [74] and Albert Cancellation Test [75] showed no sign of visual hemineglect. According to the assessments with the Chinese version of Western Aphasia Battery, the patient was not aphasic; his Aphasia Quotient was 97.8 (cutoff: 93.8). He scored 27 on mini-mental state examination (MMSE, cutoff: 24).

Two healthy men (both were 80 years old and with approximately the same educational experience with KY) served as normal controls in the behavioral experiments.

The study was approved by the Ethics Committee of the First Affiliated Hospital of Nanjing Medical University. The patient and the normal controls gave written informed consent prior to their participation in the study according to the Declaration of Helsinki, and they also provided written informed consent (as outlined in the PLoS consent form) to publication of their case details.

### Lesion analysis

In addition to the clinical MRI films taken in 2004, we acquired high contrast and high resolution structural and DTI images of the patient's brain to more accurately evaluate the lesions, particularly the splenium of the corpus callosum and occipitotemporal regions. Three types of images were acquired on a 1.5 Tesla Signa Imager (GE, Milwaukee, USA). (1) T1-FLAIR: 23 axial images were acquired with the following parameters: TR = 2019.3 ms, TE = 25.3 ms, flip angle = 90°, field of view = 240×240 mm, matrix = 256×256, slice-thickness = 5 mm, gap = 0 mm. (2) SPGR: 116 axial images, covering total cerebrum and most cerebellum, were collected with the following parameters: TR = 25 ms, TE = 3.8 ms, flip angle = 20°, field of view = 240×240 mm, matrix = 256×256, slice-thickness = 1 mm, gap = 0 mm. (3) DTI images: 35 axial diffusion-weighted single-shot spin-echo echo planar imaging (SE-EPI) were acquired with the following parameters: TR = 9000 ms, TE = 79.7 ms, field of view = 240×240 mm, matrix = 96×96, slice-thickness = 3 mm, gap = 0 mm, in-plane resolution = 0.94×0.94 mm, 15 gradient directions, b-values = 0 and 1000 s/mm^2^.

Diffusion tensors, fractional anisotropy (FA), and fiber tracts were calculated using volume-one 1.64 and diffusion tensor visualizer II (dTV II) software (Department of Radiology, Tokyo University School of Medicine). Standard methods for FA exclusion and tracking algorithms were used with a minimum FA of 0.18 and maximum angle of 30°. We identified the splenium on the mid-sagittal plane [76] and used it as an ROI (region of interest) to reconstruct fiber tracts going through the splenium.

**Figure 1:**
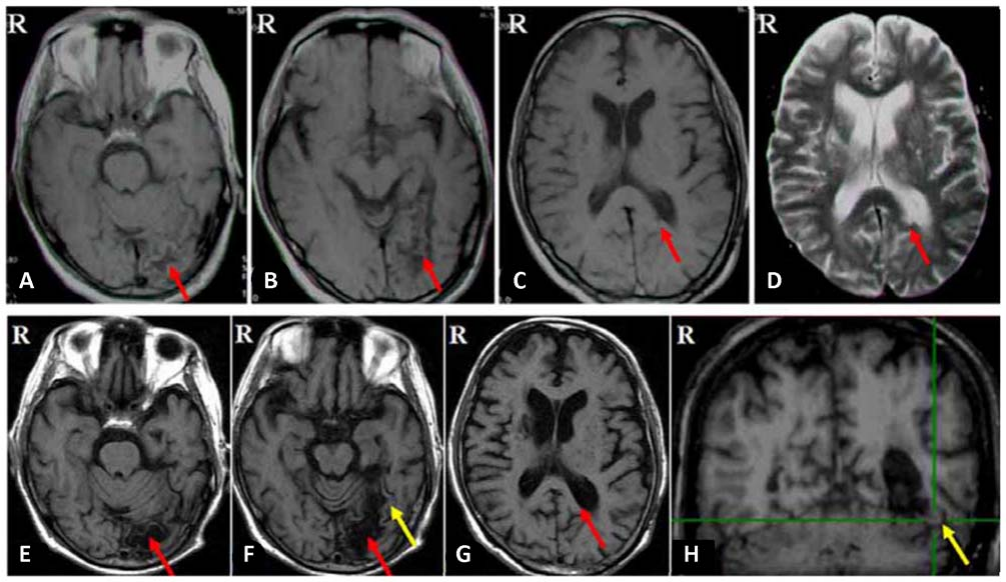
Lesions and intact VWFA shown in the structural MRI of the patient's brain. The red arrows indicate the infarctions in the left ventral medial occipitotemporal cortex (A, B, E and F) and the left splenium of the corpus callosum extending to the left major forceps (C, D and G). The yellow arrow indicates the intact left lateral mid-fusiform cortex and occipitotemporal sulcus (F and H). The VWFA is highlighted with the green crosshair (Talairach coordinates: x = −43, y = −54, z = −12) in a coronal slice of spatially normalized SPGR images (H).

**Figure 2:**
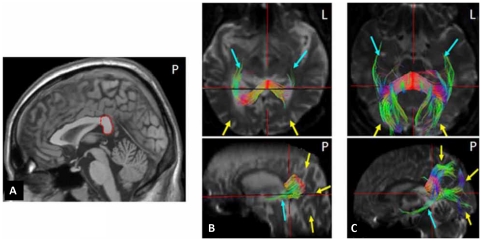
DTI fiber tracking images of the splenium of the patient and a normal control. The fiber tracking was calculated based on an ROI (marked with red circle) covering the splenium in the midsagittal plane (A). B and C show the fiber tracts through the splenium in the patient and a healthy control respectively. The yellow arrows indicate the major forceps fibers and the light blue arrows indicate the tapetum.

SE-T1 weighted (Figure 1, A, B, C) and SE-T2 weighted (Figure 1, D) axial MRIs, T1-FLAIR (Figure 1, E, F, G ), SPGR (Figure 1, H), and DTT (Figure 2) converge to indicate that the patient had infarctions in the left ventral medial occipitotemporal cortex, involving most parts of the left lingual gyrus, cuneus gyrus and fusiform gyrus, and the left splenium of the corpus callosum, extending to the left major forceps (Figure 1, C, D and G).

Note that the left lateral mid-fusiform region, medial to the left occipitotemporal sulcus, was preserved (Figure 1, F, H). This region corresponds precisely to the location of the VWFA in the literature [63], [77].

Relative to the MRIs taken 3 years ago (Figure 1, C, D), the T1-FLAIR images (G) showed, due to liquefaction of the infarction, the left major forceps became thinner and the posterior horn of left lateral ventricle became slightly larger.

The T2 weighted images (Figure 1, D) clearly show the infarction in the splenium. The fiber tracking analysis further delineated that the white matter fiber tracts going through the splenium was interrupted (Figure 2, B), whereas the tracts in the normal control was intact.

### Behavioral experiments

#### Experiment 1

**Figure 3:**
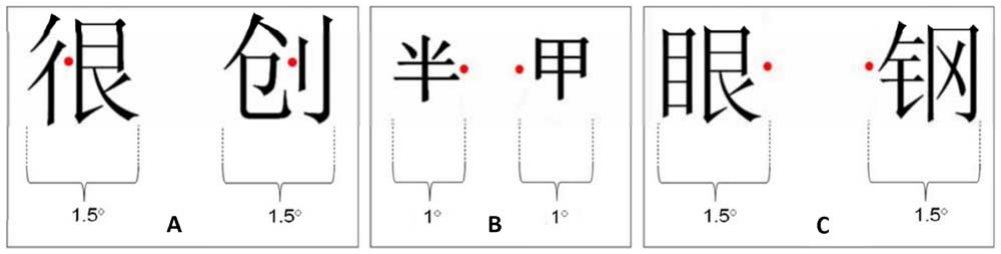
Examples of compound and simple characters used in Experiment 1 and Experiment 2. A. Example of the two types of left-right structured Chinese characters presented centrally to the foveal field (behavioral Experiment 1.). Note that the left and right components were presented completely within the left and right halves of the foveal field; the red dot between the left and right components is the fixation point. B. Divided visual field presentation of simple characters (behavioral Experiment 2). Note that the whole character was presented either within the left or right foveal field; the red dot is the fixation point. C. Divided visual field presentation of compound characters (behavioral Experiment 2). Note that the whole character was presented either within the left or right foveal field; the red dot is the fixation point.

Sixty left-right structured Chinese characters (subtended about 1.5 degree of visual angle) were presented centered at the fixation, each for 180 ms. To ensure that the left and right components of a character were projected onto right and left half of the fovea respectively, the patient was required to fixate continuously to a fixation point at the middle between the left and right components and read aloud each character (Figure 3, A). To avoid possible attentional bias caused by the differences of complexity between the left and right components (see Note 1 and [42]), we used equal numbers of SP characters (the semantic radical on the left and the phonetic radical on the right) and PS characters (the phonetic radical on the left and the semantic radical on the right), each type consisted of 30 characters. There was no difference between the two types of characters with respect to the frequency (Language and Teaching Institute of Beijing Linguistic College, 1986) and complexity (measured as the number of strokes) of the whole characters (*p*>0.05, for both measures).

Take into consideration the prevalence of homophones in Chinese characters, in Experiment 1 and 2, the patient was asked to make a word consisting of the character he read (word forming), to confirm whether the patient recognized the characters correctly or incorrectly (to disambiguate from possible homophone character). For example, after the patient read the character 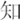 (zhi1/know)with “zhi1”, he was required to make a word with the character he recognized. If he said “zhi1 dao4” (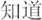/know), it would be coded as correct. If he said “zhi1 bu4” (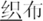/weaving), although the same pronunciation (zhi 1), it would be coded as an error. Since KY could made words correctly from characters he heard or he wrote, it is very unlikely that he made words incorrectly if he read characters correctly.

#### Experiment 2

In order to control for the possible neglect of the left or right components when they were simultaneously presented in the left and right foveal fields in Experiment 1, we used 36 simple characters (consist of one component) and 128 left-right structured compound characters in this experiment. These characters, matched for frequency and complexity, were presented randomly (each for 180 ms) in the LFF or RFF. Each simple character subtended about 0.8 degree of visual angle with the external edge at 1.0 degree to the fixation point, and each compound character subtended about 1.3 degree of visual angle with the external edge at 1.5 degree excentric to the fixation point (Figure 3, B, C). The patient was asked to fixate at the fixation point continuously and read aloud each character.

#### Experiment 3

**Figure 4:**
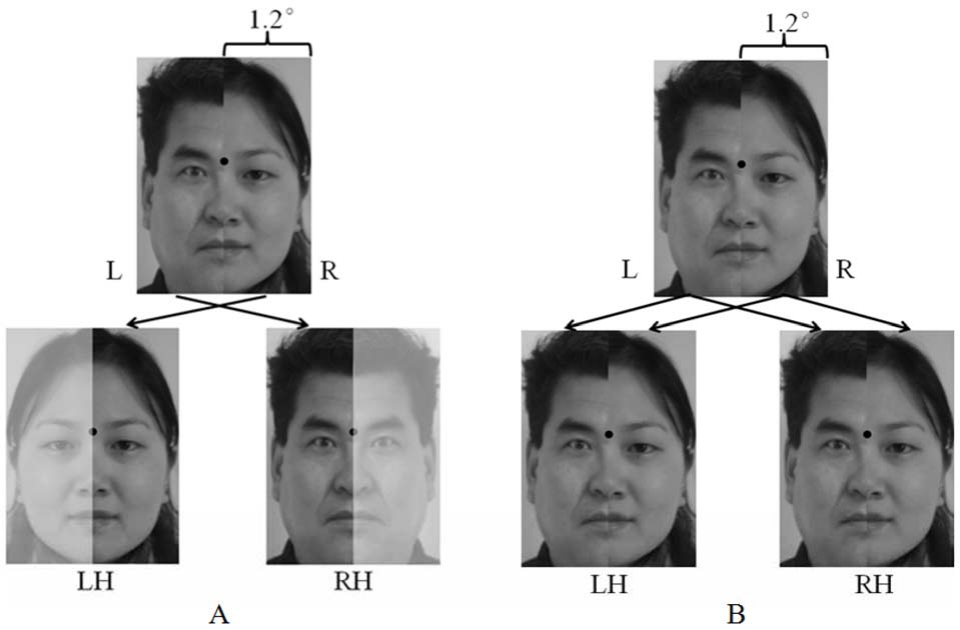
An example of the chimeric faces and its possible representations in the patient's two hemispheres. A. According to SFT, the information of the left half of the chimeric face (left to the fixation, half of a female face, upper panel) is exclusively projected to the right hemisphere (RH) and that of the right half of the chimeric face (right to the fixation, half of a male face, upper panel) is exclusively projected to the left hemisphere (LH). Because face recognition is right hemispheric dominant, the patients with interrupted splenium of the corpus callosum would make a gender judgment by the left part of the chimeric face (in this case, the patient would judge the chimeric face as a whole female face, owing to “hallucinated completions” of faces. [ Levy, Trevarthen and Sperry, 1972; Trevarthen and Kinsbourne, 1972] ). B. According to BPT, the information of the left half as well as right half of the chimeric face (upper panel) are projected simultaneously to both RH and LH. The patient would see both parts of the chimeric face.

This experiment aimed at exploring whether a face (a stimulus type rarely tested in the foveal representation domain) is double-projected to the bilateral visual cortices when it is presented in the foveal field. Twenty chimeric faces were used, 10 of them with male half-face on the left and female half-face on the right, and the other 10 were aligned conversely. Each half of a chimeric face subtended no more than 1.2 degree of visual angle (Figure 4). Each chimeric face was presented for 180 ms. The patient was asked to fixate continuously at the fixation point, which appeared at the middle of the chimeric face, and to report whether the stimulus was a male or a female face [66]. He was also asked to rate his confidence about his decision at three levels: high, modest, and no confidence.

### Statistical analysis

For behavioral results, the key comparison was on the difference between the LFF and RFF condition in response accuracy for recognizing characters and components as well as chimeric face gender judgment. The statistical significance of these differences was tested with Chi-Square test (SPSS, Version 11.5 ).

### fMRI experiment

This experiment was similar to that used by Cohen et al. [63]. However, there were important differences between two studies. First, Chinese compound characters rather than English words were adopted in our study. Second and more importantly, all stimuli were presented within the foveal field (<1.2 degree eccentricity) in this experiment, but the mean eccentricity of the stimuli was 4.9°, i.e., outside the foveal region, in the study of Cohen et al. [63].

#### Stimuli

The stimuli were 80 left-right structured compound characters (each consisted of 2 to 4 radicals and 6 to 11 strokes). All characters were highly imaginable common nouns with high frequency. These characters were grouped into two lists, 40 characters each, matched one-to-one for the numbers of strokes and radicals. The two lists were also matched for overall frequency. Each list was further divided into four sets, 10 characters for each. Two sets, one from each list, formed one pair. Two sets characters of the same pair were matched one-to-one for the numbers of radicals and strokes and overall frequency. By doing so, we created four pairs (8 sets: a and a′, b and b′, c and c′, d and d′) of stimuli, and each set consisted of 10 characters.

#### Stimulus parameters, task, and procedures

**Figure 5:**
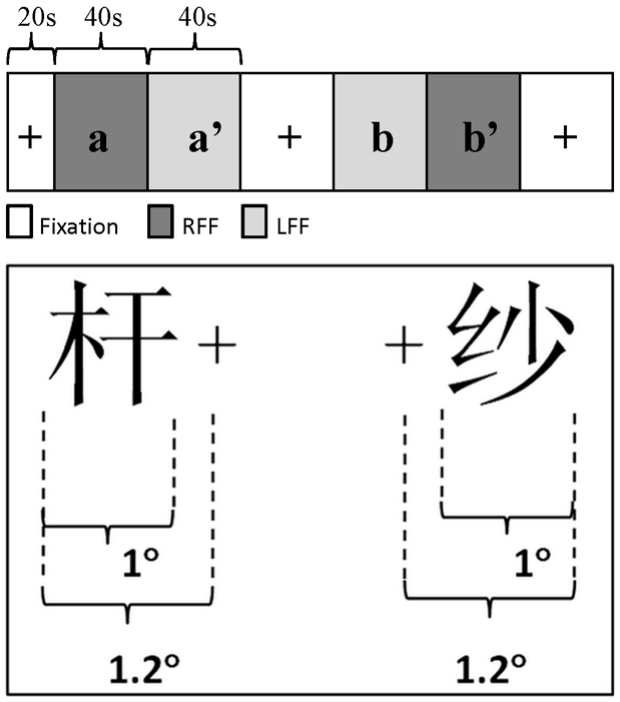
Schematic depiction of the fMRI experimental paradigm and examples of two types of stimuli. Upper panel: fMRI experimental paradigm (only the first run is shown); lower panel: examples of two types of stimuli presented in the LFF or RFF. +: fixation point; a/a′ and b/b′: two paired characters lists, matched one-to-one for the numbers of strokes, radicals, and overall frequency.

Four fMRI runs were performed; each consisted of an alternation of activation blocks (40 s) and fixation blocks (40 s), starting with a 20 s fixation epoch to allow T1 equilibration (Figure 5). During the activation blocks, Chinese characters flashed either into the patient's RFF or LFF. There were 10 trials in each activation block. Each trial began with a fixation epoch of 3820 ms and followed by a presentation epoch, in which each character was presented for 180 ms. The characters were shuffled randomly within each set of 10 trials (a block). Each character appeared twice, once in the RFF block and once in the LFF block. The medial and lateral edges of each character were 0.2° and 1.2° from the fixation point respectively (Figure 5). The patient was asked to fixate at a continuously present central cross-hair and to read characters silently whenever he saw the characters on the screen.

#### Imaging acquisition and analysis

23 axial functional images were acquired with a T2*-weighted gradient-echo, echo planar imaging (EPI) sequence on the GE 1.5 Tesla scanner [TR = 2000 ms, TE = 40 ms, flip angle = 90°, field of view = 240×240 mm, thickness = 5 mm, gap = 0 mm, in-plane resolution = 3.75×3.75 mm, 120 volumes for each run]. T1-FLAIR and 3D- SPGR Image were also acquired for anatomical localization (see the Lesion Analysis section).

The AFNI package (Cox 1996, http://afni.nimh.nih.gov/afni/) [78] was used for image display and data analysis. The first 10 volumes in each run were discarded, leaving a total of 480 EPI volumes that were analyzed for brain activation. The 3D-SPGR data was normalized to the standardized space of Talairach and Tournoux [79]. Functional MR images were motion-corrected and smoothed with an isotropic Gaussian kernel (FWHM = 3 mm) to enhance the signal-to-noise ratio. Using the 3-D deconvolution program of AFNI, the impulse response function for each condition was estimated and multiple regressions were calculated for each voxel to test the fitness between the observed time series and the estimated response. Those voxels whose F values were equal to or greater than threshold (*P* = 0.05, corrected (FDR)) were defined as task-relevant, and were superimposed on the anatomic images to produce activation map for each condition.

## Results

### Behavioral experiments

The results of three behavioral experiments are summarized in Table 1.

**Table 1:** Summary of the results of three behavioral experiments.

	Experiment 1	Experiment 2		Experiment 3
	Reading			Gender judgment
	compound characters in the central fovea	simple characters in the LFF/ RFF	compound characters in the LFF/ RFF	Chimeric face in the central fovea
Whole characters	14/60(23%)	—	—	—
Components (Characters) in LFF (Correct rate, %)	14/60(23%)	8/18(44%)	0/64(0%)	—
Components (Characters) in RFF (Correct rate, %)	44/60(73%) 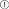	17/18(94%) 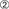	33/64(52%) 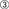	—
Judged as the gender of the left half face (%)	—	—	—	17/20 (85%)
Judged as the gender of the right half face (%)	—	—	—	3/20 (15%) 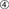

Note: LFF = left foveal field, RFF = right foveal field; * LFF *versus* RFF,

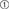
x^2^(1) = 30.0, p = 0.000;

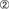
x^2^(1) = 10.6, p = 0.003;

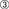
x^2^(1) = 44.5, p = 0.000;

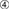
x^2^(1) = 19.6, p = 0.000.

#### Experiment 1

This experiment was designed to examine the patient's reading performance when Chinese compound characters were briefly presented in the central foveal field. In this condition, the patient could correctly read out only 14 of 60 characters tested (the accuracy for the PS characters was not significantly different from that for the SP characters; we therefore pooled together the two types of characters in the subsequent analyses). Among the 46 characters he could not correctly read, 38 were misread and 8 were unrecognized. His reading problem was mostly resulted from his difficulty in identifying the left components. Indeed, among the 38 characters he misread, two were misread because of the omission of the left components (he read the right components as the names of the characters), For example, he read 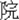 (/yuan 4/, yard) as 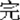 (/wan 2/, finish) and read 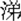 (/ti 4/, tears) as 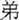 (/di 4/, brother). 26 were substituted by different characters that contain the same right components but with different left components as the tested characters. In other words, the left components of these 26 characters were substituted rather than omitted. For example, 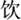 (/yin 3/, drink) was misread as 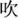 (/chui 1/, blow), 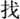 (/zhao 3/, search) as 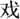 (/xi 4/, game). The remaining 10 characters were recognized as characters with similar shape, such as 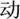 (/dong 4/, move) as 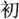 (/chu 1/, beginning), 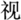 (/shi, 4/, watch) as 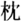 (/zhen 3/, pillow).

For KY, the correct rates of recognition of the left and right component were 14/60(23%) and 44/60(73%) respectively. The difference was significant (x^2^(1) = 30.0, p = 0.000 ) (Table 1). The two normal controls correctly recognized all the tested stimuli.

#### Experiment 2

This experiment was to test the patient's performance of reading whole characters presented entirely within the LFF or RFF. Relative to the RFF condition, he showed much severer deficits in reading aloud and recognizing the characters that were presented in the LFF (simple character: x^2^(1) = 10.6, p = 0.003; compound characters: x^2^(1) = 44.5, p = 0.000), with the scores for simple characters being slightly higher than for the compound characters (see Table 1). This difficulty in reading characters in the left visual field was consistent with left hemialexia, reported in alphabetic languages [63], [64], [80]. The patient also showed a symptom of perseveration when he read compound characters presented in the LFF. For example, he misread more than 10 characters as the same character, i.e., 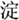 (/dian 4/, sediment), in a stereotyped manner.

The two normal controls correctly recognized and read aloud essentially all these simple and compound characters. One subject made no mistake at all, and one subject made a single mistake, misreading 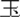 (/yu 4/ jade) (presented in the RFF) as 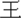 (/wang 2/king).

#### Experiment 3

In this experiment, we attempted to provide a complementary test independent of reading for the two theories on cortical representation of foveal vision. As shown in Table 1, in the task of gender judgment of chimeric faces, the patient made gender decisions mostly on the basis of the left half faces in the LFF (17/20), whereas only 3/20 was based on the right half faces in the RFF(x^2^(1) = 19.6, p = 0.000). Importantly, he was fairly confidence about his judgments in all 20 chimeric faces (high confidence: 6, modest confidence: 14, no confidence: 0), i.e., he was virtually not aware that the faces were chimeric (see Figure 4A, lower panel).

In contrast, the two normal controls were aware that all face stimuli were made of two half faces of opposite genders.

### fMRI results

Overall, Chinese characters activated a distributed network, involving bilateral dorsolateral prefrontal, medial frontal, and cingulate cortices, whereas additional activations were found in the left fusiform, parietal gyri, under the RFF presentation condition.

**Figure 6:**
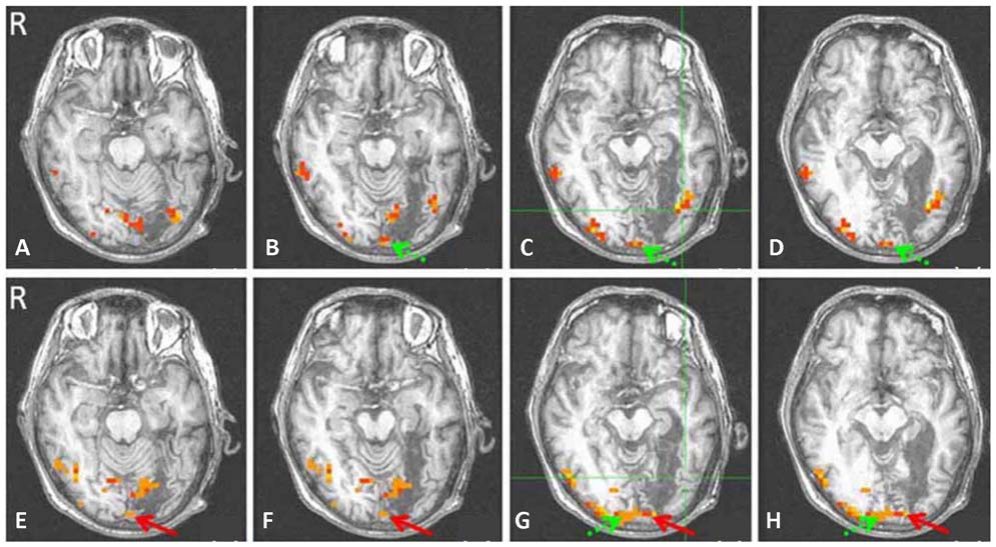
fMRI activation maps for two presentation conditions in the patient. Upper: Characters presented in the RFF, Lower: Characters presented in the LFF. The VWFA in the left lateral mid-fusiform cortex was activated when Chinese characters were presented in the RFF(C, the green crosshair) but not in the LFF (G, the green crosshair). The Characters in the RFF activated only the left early visual cortices (green arrow in B, C, and D). However, both the right (green arrow in G, H) and left early visual cortices (red arrow in E-H, at the same axial level as A-D respectively) were activated when the Characters were presented in the LFF.

As clearly shown in Figure 6 (upper, C), when Chinese characters were presented in the RFF but not the LFF, a region in the left lateral mid-fusiform cortex was significantly activated(*P*≤0.05, FDR-corrected). This region (Talairach Coordination: peak voxel: x = −38, y = −65, z = −1; extent: x = −35∼−47; y = −56∼−68; z = −19∼3; Cluster: 45 voxels) corresponds precisely to the location of the VWFA in the literature [63], [77].

Interestingly, there is some evidence for bilateral activation(*P*≤0.05, FDR-corrected) in the early visual cortex from stimuli presented in the LFF (Figure 6, lower, green and red arrow, Talairach Coordination: x = 14, y = −71, z = −1, Cluster: 9 voxels; x = 26, y = −83, z = 18, Cluster: 7 voxels; x = −23, y = −71, z = −10, Cluster: 13 voxels), however the stimuli in the RFF only activated the left early visual cortex (Figure 6, upper, green arrow, Talairach Coordination: x = −11, y = −83, z = −10, Cluster: 18 voxels; x = −26, y = −83, z = −10, Cluster: 32 voxels).

## Discussion

The main goal of this study was to assess the two hotly debated theories, the BPT and SFT, in a unique patient with splenium lesion, by the combined use of neuropsychological tests and multimodal imaging techniques (including T1/T2 weighted structural MRIs, fMRI, and DTI).

Throughout our study, we have carefully considered the following three critical methodology issues (see [29], [30], [35], [54], [81] for detailed discussion). First, inter-hemispheric information transfer and inhibition should be avoided or controlled for. Accordingly, in the current study, we recruited a unique patient with a splenium lesion, which was confirmed by multimodal MRIs, particularly the DTI analysis. Second, the stimuli should be presented precisely within the foveal field even in the half foveal field. In this study, all stimuli, including reading materials and chimeric faces, were presented within the foveal field. In addition, as discussed in the Introduction, the visual features of Chinese characters allow us to present them more easily, relative to alphabetic words, within the foveal field. Third, more than one type of visual materials should be used, ideally with the combined use of behavioral and biological methods. We thus adopted not only a series of reading tasks but also a face recognition task. Although several authors have pointed out the importance of face stimuli in understanding the nature of foveal representation [30], [35], to our knowledge, no such a study has yet been reported. Importantly, our fMRI experiment revealed the physiological manifestations of visual perception at both higher and lower levels in the same individual, showing a more comprehensive picture of foveal representation.

Results of the three behavioral experiments and fMRI activation in the VWFA only from RFF stimulation provide converging support for the SFT, although the bilateral activations of the early visual cortices under the LFF presentation conditions seems to be consistent with the BPT. We next discuss these seemingly conflicting findings together with the relevant results of the literature.

Behavioral experiment 1 demonstrated that the patient had difficulties in recognizing reading materials presented in the foveal region but left to the fixation. Specifically, when the left-right structured compound Chinese characters were presented briefly in the central foveal field, he could correctly read only 14 out of the 60 characters tested. Critically, his reading errors were mainly resulted from the failure of recognizing the ***left*** components of these compound characters, in that he could recognize the right components of 73% characters tested but the left component only 23% of characters tested. The patient's above reading problems is unlikely caused by his attentional bias to the right components (see Note 1), because he made virtually the same number of errors in reading both types of the characters.

Another possible interpretation was his neglect of the left components, particularly when left and right components were simultaneously presented in the LFF and RFF. However, this interpretation is inconsistent with the fact that for the characters the patient misread, he misread rather than omitted the left components. Specifically, among the 38 characters he misread, in only 2 of them the left component was omitted (he read instead the character with the name of the right component), whereas in 26 of the misread characters, the left components were substituted by different components.

The possibility of neglect is further ruled out by the patient's performance in the two neglect tests and behavioral Experiment 2. His performances were normal in the line bisection test [74] and the cancellation task [75], the two most commonly used clinical tests for neglect. In behavioral Experiment 2, the whole characters were randomly presented in the LFF or RFF (thus no extinction), and the patient was asked to read them aloud. When the stimuli were presented in the LFF, he read correctly 8 of 18 simple characters and none of 64 compound characters. When the stimuli were presented in the RFF, however, his reading performance was reasonably good: he correctly read 17 of 18 simple characters and 33 of 64 compound characters.

The behavioral Experiment 3, which did not involve reading, adds another piece of evidence for the SFT. In this experiment, the patient was asked to perform a facial gender decision task, in which the chimeric face (half female face and half male face) was presented strictly within the foveal field. As Table 1 clearly shows, his judgment for the gender of the chimeric was virtually (17 out of 20) exclusively based on the left half-face while ignoring the right half-face. This result is well in accordance with the SFT. First, the patient's right fusiform was intact and numerous studies have demonstrated a right hemispheric dominance for processing face information [66]–[71]. Second, although some studies suggest that in the left fusiform there is a face processing area (the left FFA), medial to the VWFA [72], [82]–[83], our detailed lesion analysis revealed that the patient's left FFA might be damaged while his VWFA remained intact. Therefore, his facial gender judgment was likely dependent on the (right) FFA, which is consistent with the observation that the patient was not aware at all that the faces he was viewing were chimeric.

More direct evidence for SFT comes from our fMRI experiment. As shown in Figure 6, the Chinese characters presented in the RFF significantly activated the left lateral mid-fusiform gyrus (upper, c, the green crosshair), but those presented in the LFF did not. The left lateral mid-fusiform gyrus, i.e., VWFA, has been well documented as a critical locus for visual word processing [63], [65], [77], [83]–[86]. Our fMRI results are consistent with those of Cohen and colleagues' studies in splenium lesion patients [63], [64]. However, in these two studies, written words were presented in eccentricities of 4.9° [63] and 2°–6° [64] respectively, well beyond the foveal field, thus their results could not be used to address the issue related to SFT and BPT, whereas our stimuli were presented completely within the foveal field (≤1.5°).

Thus, both behavioral and fMRI results we discussed so far are in favor of the SFT but against the BPT.

However, our fMRI data also showed activation in both sides of the early visual cortices when the stimulus was presented in the LFF. This finding could be considered as evidence for the BPT, which is also consistent with results of a large number of previous studies [1]–[18], [81], [87]. For example, Bunt et al. [3] provided anatomical evidence for bilateral foveal projections in the monkey, although there were only about 7% (1/14) ganglion cells projecting to opposite dLGN (dorsal lateral geniculate nucleus). Frendich et al. [13] demonstrated behavioral evidence for double, although weak, projections in a callosotomy patient. Vitctor et al. [87] and Magni et al. [88] observed bilateral cortical representations of the foveal information, both using visual evoked potentials. Kraft et al., [21] found bilateral activations in early visual areas (V1–V3/Vp) when the stimuli (e.g., checkerboards or colored objects, subtending 1.4° of visual angle) were presented 1.2 degree of visual angle right or left from the vertical meridian.

Therefore, based on the above finding, a clear picture emerges regarding the cortical representation of fovea vision. We believe that the foveal region does have bilateral projection to the early visual cortex, much more robust contralaterally than ipsilaterally, this account for the foveal sparing as well as other evidence that supports BPT. However, although the ipsilateral projection may show in fMRI studies of early visual cortex, and in perimetry measures of simple visual detection, at object processing levels, our study show that the representation of foveal stimuli is functionally effectively split. This account accommodates the key evidence for BFT, yet the fundamental conclusion is that foveal vision is functionally split, which agrees with the SFT and is consistent with a large number of behavioral studies involving object processing, including reading and object naming [24]–[48].

In conclusion, the present study investigated a unique patient with lesions in the splenium of the corpus callosum and the left medial occipitotemporal region, through a series of neuropsychological tests and multimodal MRI scans. Results of the current study, together with existing evidence in the literature, suggest that the representation of foveal stimuli, although likely bilaterally projected in early visual cortices, is effectively functionally split at object processing levels.
